# Exploring the Molecular Targets for the Antidepressant and Antisuicidal Effects of Ketamine Enantiomers by Using Network Pharmacology and Molecular Docking

**DOI:** 10.3390/ph16071013

**Published:** 2023-07-17

**Authors:** Glorister A. Altê, Ana Lúcia S. Rodrigues

**Affiliations:** Department of Biochemistry, Center of Biological Sciences, Federal University of Santa Catarina, Florianópolis 88037-000, SC, Brazil; glorister.alte@ufsc.br

**Keywords:** esketamine, arketamine, bioinformatical analysis, depression, suicide

## Abstract

Ketamine, a racemic mixture of esketamine (S-ketamine) and arketamine (R-ketamine), has received particular attention for its rapid antidepressant and antisuicidal effects. NMDA receptor inhibition has been indicated as one of the main mechanisms of action of the racemic mixture, but other pharmacological targets have also been proposed. This study aimed to explore the possible multiple targets of ketamine enantiomers related to their antidepressant and antisuicidal effects. To this end, targets were predicted using Swiss Target Prediction software for each ketamine enantiomer. Targets related to depression and suicide were collected by the Gene Cards database. The intersections of targets were analyzed using Gene Ontology (GO) and Kyoto Encyclopedia of Genes and Genomes (KEGG). Network pharmacology analysis was performed using Gene Mania and Cytoscape software. Molecular docking was used to predict the main targets of the network. The results indicated that esketamine and arketamine share some biological targets, particularly NMDA receptor and phosphodiesterases 3A, 7A, and 5A but have specific molecular targets. While esketamine is predicted to interact with the GABAergic system, arketamine may interact with macrophage migration inhibitory factor (MIF). Both ketamine enantiomers activate neuroplasticity-related signaling pathways and show addiction potential. Our results identified novel, poorly explored molecular targets that may be related to the beneficial effects of esketamine and arketamine against depression and suicide.

## 1. Introduction

Major depression disorder (MDD) is the leading cause of disability and the largest contributor to suicide deaths in the world [1]. According to the WHO (2017) [2], every year, about 800,000 individuals take their own lives. Chronic stress events, such as the COVID-19 pandemic, have been linked to the development of depression and anxiety, as well as suicidal ideation [3]. The pathophysiology of MDD is complex and multifactorial, involving the functioning of several neurotransmission systems besides monoamines. The modulation of neuroplasticity events, particularly those related to BDNF signaling, and the process of neuroinflammation are events closely implicated in the development of depressive symptoms [4,5]. Despite distinct molecular mechanisms, suicide attempts (SA) and suicidal ideation demonstrate a shared genetic etiology with MDD, and severe depressive symptoms play a crucial risk factor [6,7].

Ketamine is a racemic mixture of S-ketamine (esketamine) and R-ketamine (arketamine) that has been used due to its anesthetic and analgesic properties since the 1970s. Recently, esketamine was approved by the FDA for the treatment of refractory depression and suicidal ideation because of its rapid, potent, and sustained effect, which differs substantially from the antidepressant profile of conventional antidepressants. The pharmacological properties of ketamine are mainly attributed to the inhibition of N-methyl-D-aspartate receptors (NMDAr). However, ketamine has multiple pharmacological targets, such as gamma aminobutyric acid (GABA), dopamine, serotonin, opioids, and cholinergic receptors [8]. A single subanesthetic dose of nasal esketamine produces rapid and long-lasting effects in patients resistant to other antidepressants [9]. Evidence indicates that the rapid antidepressant effect of ketamine may involve antagonism of NMDAr on GABAergic interneurons, preventing the inhibition of glutamatergic tone, as proposed by the disinhibition hypothesis [8,10]. Consequently, AMPA receptors are activated, causing cell depolarization and the activation of voltage-dependent cell-gated channels. Calcium entry then stimulates the exocytosis of synaptic vesicles containing BDNF, which causes the activation of TrKB receptors, culminating in the activation of mTOR complex 1 (mTORC1) [8,10,11]. 

One possibility to study the multitarget potential of ketamine is through bioinformatics tools, which allow screening of depression and suicide molecular targets rapidly and in an early approach that can guide future higher cost experimental studies. Bioinformatics is an area with great potential applications for the study of diseases and drug design and discovery. Pharmacological target network analysis, molecular docking, and gene ontology (GO) enrichment analyses may allow the understanding of the polypharmacological action of molecules, investigating their potential targets, and allowing a more rational use of animal models in scientific research [12,13,14,15,16]. Computational approaches are already used by the pharmaceutical industry for the development of new drugs, because besides the economic advantage, they have the potential to accelerate discoveries [17,18,19]. 

In this study, we used some of these bioinformatics tools to investigate the potential molecular targets of ketamine enantiomers in depression and suicide. Ketamine has been reported to exhibit polypharmacological action, and there may be differences between esketamine and arketamine in terms of their potency and biological effects, which may be due to their ability to interact with different molecular targets [20,21]. Therefore, we also computationally investigated the overlapping and/or unique molecular targets of esketamine and arketamine in depression.

## 2. Results

### 2.1. Identification of Ketamine Enantiomers, Suicide, and Depression—Overlapping Genes

According to the SwissTargetPrediction database, 105 potential targets of arketamine and 105 potential targets of esketamine were found. Importantly, although most of the target genes were shared by the enantiomers (91 genes), there were differential genes that were unique targets for arketamine (14 genes), and others unique to esketamine (14 genes).

As a result of the search for depression-related genes and suicide-related genes in the GeneCards database, we obtained a total of 13,914 genes and 2409 genes, respectively. Of note, suicide and depression shared 1983 genes, according to the prediction. 

**Figure 1:**
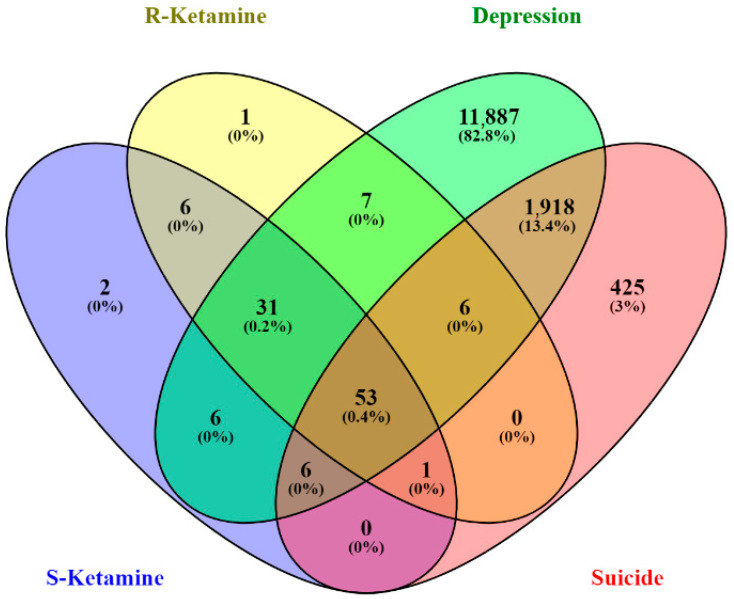
Venn diagram showing potential target genes related to depression, suicide, and ketamine enantiomers (R and S).

The Venn diagram illustrated in Figure 1 shows potential targets among depression-related genes, suicide-related genes, and esketamine- and arketamine-related genes. The intersection of arketamine and esketamine and depression- and suicide-related genes yielded 53 potential targets. The Venn diagram also showed 31 genes intersected by depression, arketamine, and esketamine, 6 genes intersected by depression, suicide, and arketamine (*PDE4B*, *KDM4C*, *MIF*, *NOS2*, *CREBBP*, *HMOX1*), and 6 genes intersected by depression, suicide, and esketamine (*SIRT1*, *PIK3CA*, *ICAM1*, *GABRA1*, *GABRB2*, *PRF1*). According to the Venn diagram, no overlapping genes were observed between arketamine and suicide or between esketamine and suicide. However, the *CA12* gene that codes for carbonic anhydrase 12 was shared by esketamine, arketamine, and suicide. Considering that ketamine has been used for treating severe depression with suicide ideation [8], we focused on depression and suicide overlapping genes for network analyses.

#### 2.1.1. Network Construction of Ketamine Enantiomers, Suicide, and Depression Overlapping Genes

**Figure 2:**
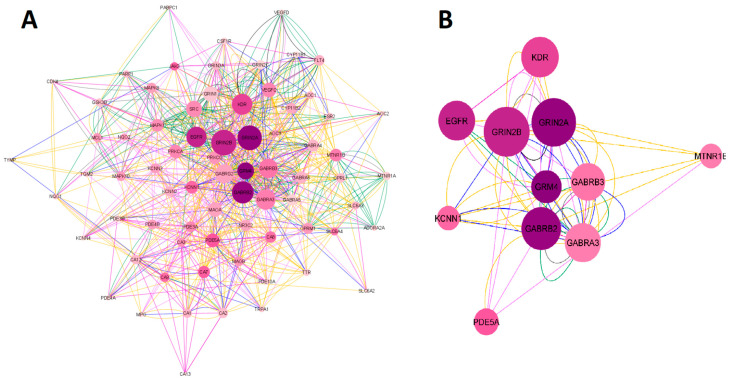
Network construction of esketamine, arketamine, suicide, and depression overlapping genes. (**A**) Complete network. (**B**) The filtered network based on the degree center (DC) > 30 and betweenness centrality (BC) range of 0.017–0.044. The size of the nodes represents the degree of the node. The color of the edges represents the types of interactions. Physical interactions are represented in green, co-expression in yellow, shared protein domains in pink, co-locations in blue, predictions in gray, genetic interactions in purple, and metabolic pathways in black. This network shows the 11 most central and connected target genes shared among ketamine enantiomers, suicide, and depression.

The search for the ketamine enantiomers, suicide, and depression overlapping genes in *Homo sapiens* was performed in GeneMANIA. The obtained network was imported and analyzed in Cytoscape V. 3.9.1. software (The Cytoscape Consortium, San Diego, CA, USA). As a result, a network with 73 nodes, 891 connections, and a clustering coefficient equal to 0.349 was obtained. The colors of the edges (connections) reflected the type of interaction analyzed. The network had 891 connections, of which 34.85% were physical interactions (green), 28.20% were co-expressed (yellow), 25.94% represented shared protein domains (pink), 9.09% were co-localized (blue), 7.20% were predictions (gray), 2.84% were genetic interactions (purple), and 1.88% were metabolic pathways (black). The network construction of esketamine, arketamine, suicide, and depression overlapping genes is shown in Figure 2A. Regarding the network organized in Cytoscape software, the larger the size of the node, the higher the degree (n° of connections) of a given protein, and the darker the color, the higher the betweenness centrality (importance of that protein as a “bridge” or shortest pathway in the network). When we built the network with 53 overlapping genes, 20 genes were retrieved in the network, namely *KCNN4*, *NQO1*, *AOC2*, *VEGFD*, *VEGFC*, *CA3*, *AOC1*, *CA12*, *GABRA3*, *PDE5A*, *MTNR1B*, *GABRB2*, *CA13*, *GRIN2C*, *CA8*, *GABRA6*, *GABRA4*, *GRIN3A*, *PDE4B*, and *PDE3B*. Subsequently, the network was filtered considering degree > 30 and betweenness centrality between 0.017 and 0.044. From these data, 11 genes were found to have the highest number of connections (Degree > 30) and the shortest paths (betweenness centrality) in the network. The genes found were *GRIN2B*, *GRIN2A*, *GRM4*, *EGFR*, *KDR*, *GABRB3*, *GABRB2*, *GABRA3*, *KCNN1*, *PDE5A*, and *MTNR1B*. The proteins encoding for these genes were as follows: NMDA receptor (*GRIN2B* and *GRIN2A*), metabotropic glutamate receptor (*GRM4*), GABA receptor subunits β2, β3 and α3 subunits (*GABRB3*, *GABRB2*, *GABRA3*), epithelial growth factor receptor (*EGFR*), melatonin receptor 1β (*MTNR1B*), kinase insertion domain receptor (*KDR*), member 1 of the N subfamily of calcium-potassium activated channel (*KCNN1*), and the enzyme phosphodiesterase 5A (*PDE5*). The filtered network of the targets obtained is illustrated in Figure 2B. 

#### 2.1.2. Network Construction of Esketamine, Suicide, and Depression Overlapping Genes

**Figure 3:**
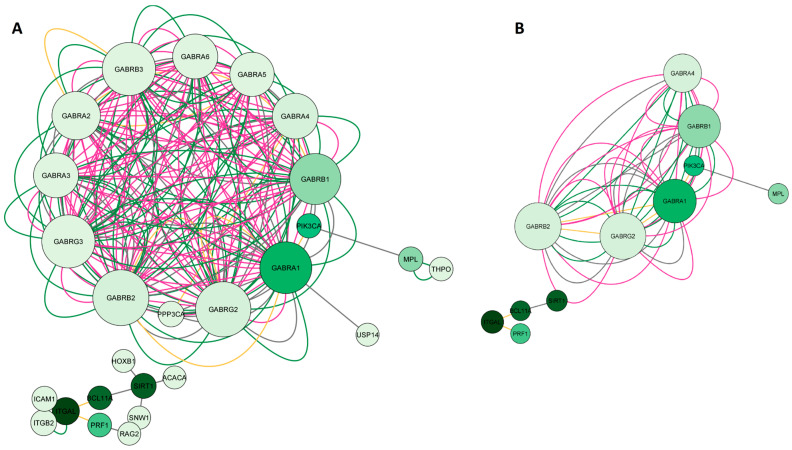
Network construction of esketamine, suicide, and depression overlapping genes. (**A**) Complete network. (**B**) The filtered network based on the degree center (DC) > 2 and betweenness centrality (BC) range of 0.012–0.0667. The size of the nodes represents the degree of the node. The color of the edges represents the types of interactions. Physical interactions are represented in green, co-expression in yellow, shared protein domains in pink, co-locations in blue, predictions in gray, genetic interactions in purple, and metabolic pathways in black. This network shows the 11 most central and connected target genes shared among esketamine, suicide, and depression.

In the second step of the network analysis, we obtained potential targets shared by esketamine, suicide, and depression. Therefore, the green diagram was built by searching for the genes *SIRT1*, *PIK3CA*, *I*-*CAM1*, *GABRA1*, *GABRB2*, and *PRF1*. As a result, we obtained a network with 26 nodes, 242 connections, and a clustering coefficient equal to 0.714. Figure 3A shows a high degree of clustering among the genes encoding GABAergic receptor subunits, which were more connected to each other than to other genes. Most interactions were physical interactions (48.92%), followed by predicted interactions (37.4%), co-expression (10.2%), and shared protein domains (3.48%). In the network construction, we retrieved 20 genes. After applying degree (DC > 2) and betweenness centrality (BC) filters between 0.012 and 0.0667, we obtained the 11 most important genes in the network, as illustrated in Figure 3B. Most of these genes encoded GABAergic receptor subunits (*GABRA1*, *GABRB1*, *GABRA4*, *GABRG2*, *GABRB2*), but genes encoding sirtuin 1 (*SIRT1*), integrin subunit alpha L (*ITGAL*), phosphatase (*FOSPI3K*) and kinase (*PI3K*) subunits, thrombopoietin receptor (*MPL*), transcription factor (*BCL11A*), and perforin 1 (*PRF1*) were also identified.

#### 2.1.3. Network Construction of Arketamine, Suicide, and Depression Overlapping Genes

**Figure 4:**
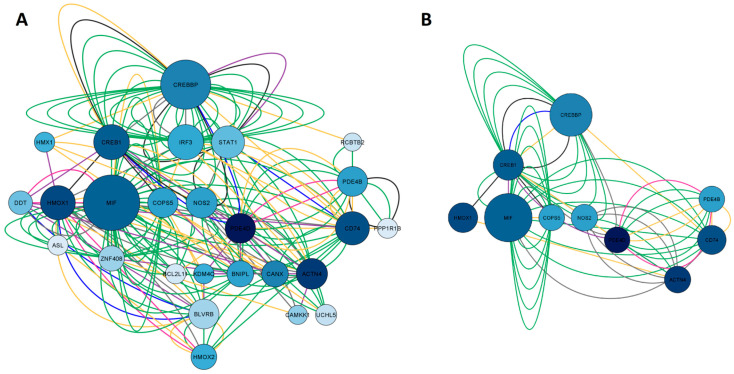
Network construction of arketamine, suicide, and depression overlapping genes. (**A**) Complete network. (**B**) The filtered network based on degree center (DC) > 16 and betweenness centrality (BC) range of 0.039–0.157. The size of the nodes represents the degree of the node. The color of the edges represents the types of interactions. Physical interactions are represented in green, co-expression in yellow, shared protein domains in pink, co-locations in blue, predictions in gray, genetic interactions in purple, and metabolic pathways in black. This network shows the 10 most central and connected target genes shared between arketamine, suicide, and depression.

The third network, represented in Figure 4A, whose nodes are in blue, represented the common targets between arketamine, depression, and suicide and was assembled based on the search for the genes *CREBBP*, *NOS2*, *HMOX1*, *KDM4C*, *PDE4B*, and *MIF*. As a result, we obtained a network with 26 nodes, 189 connections, and a clustering coefficient equal to 0.293. When we performed the pharmacological network analysis of only esketamine, the result was a cluster of GABA_A_ receptors (with a clustering coefficient equal to 0.714, where the closer to 1, the less random the nodes of a network were involved), highlighting the genes coding for some specific subunits, such as *GABRA1*, *GABRA4*, *GABRB1*, *GABR2,* and *GABRG2.* Most interactions were physical interactions (80.30%), followed by predicted interactions (9.48%), genetic interactions (4.28%), co-expression (3.72%), metabolic pathways (1.12%), co-localization (0.74%), and shared protein domains (0.37%). In the network construction, we retrieved 20 genes. After applying degree (DC > 16) and betweenness centrality (BC) filters between 0.039 and 0.157, we obtained the 10 most important genes of the arketamine, depression, and suicide network, as illustrated in Figure 4B. Most of these genes coded for signaling pathway-related proteins (*CREB1*, *CREBBP*, and *COPS5*), enzyme-related genes (*PDE4B*, *PDE4D*, *NOS2*, and *HMOX1*), genes related to immune responses (*MIF* and *CD74*), and cytoskeleton (*ACTN4*). 

#### 2.1.4. Network Construction of Ketamine Enantiomers and Depression Overlapping Genes

**Figure 5:**
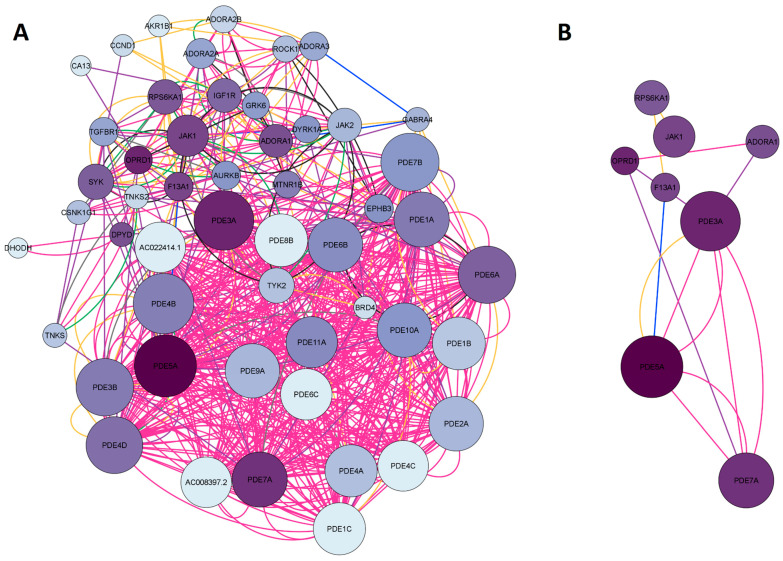
Network construction of esketamine, arketamine, and depression-overlapping genes. (**A**) Complete network. (**B**) The filtered network based on degree center (DC) > 11 and betweenness centrality (BC) range of 0.038–0.079. The size of the nodes represents the degree of the node. The color of the edges represents the types of interactions. Physical interactions are represented in green, co-expression in yellow, shared protein domains in pink, co-locations in blue, predictions in gray, genetic interactions in purple, and metabolic pathways in black. This network shows the 8 most central and connected target genes shared between ketamine enantiomers, and depression.

The purple network represented in Figure 5A was built by searching for the genes *ROCK1*, *AKR1B1*, *JAK2*, *DHODH*, *TYK2*, *PDE4C*, *TGFBR1*, *TNKS2*, *GABRA4*, *CCND1*, *PDE4D*, *OPRD1*, *RPS6KA1*, *F13A1*, *MTNR1B*, *PDE5A*, *IGF1R*, *BRD4*, *PDE3B*, *DYRK1A*, *JAK1*, *ADORA1*, *SYK*, *CSNK1G1*, *ADORA2B*, *TNKS*, *AURKB*, *EPHB3*, *CA13*, *GRK6*, and *ADORA3*, which are the 31 target genes shared by esketamine, arketamine, and depression. As a result, a network with 51 nodes, 698 connections, and a clustering coefficient equal to 0.584 was obtained. This high degree of clustering was composed of several types of phosphodiesterases. Most interactions in this network were shared protein domains (42.12%), followed by co-expression (26.24%), metabolic pathways (17.70%), physical interactions (6.43%), co-localization (5.77%), genetic interactions (0.98%), and predicted interactions (0.76%). In the network construction, we retrieved 20 genes. After applying degree (DC > 11) and betweenness centrality (BC) filters between 0.038 and 0.079, we obtained the 8 most important genes, as illustrated in Figure 5B. Most of these genes encoded phosphodiesterases (PDE5A, PDE7A, PDE3A), opioid receptor delta 1 (OPRD1), adenosine A1 receptor (ADORA1), and Janus Kinase 1 (JAK1) involved in inflammation and epithelial remodeling, a subunit A of coagulation factor XIII (F13A1) with catalytic function, and a ribosomal protein S6 Kinase A1 (RPS6KA1) related to cell growth and differentiation.

### 2.2. GO and Kyoto Encyclopedia of Genes and Genomes (KEGG) Enrichment Analysis of Ketamine Enantiomers, Suicide, and Depression Overlapping Genes

**Figure 6:**
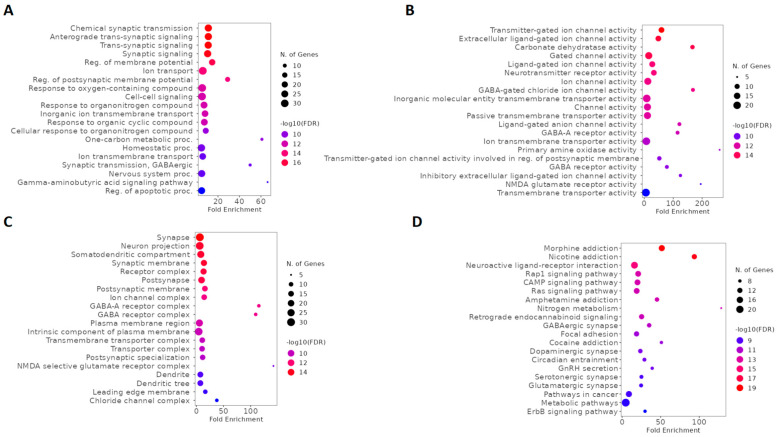
GO and KEGG enrichment analysis of ketamine enantiomers, suicide, and depression overlapping genes. The top 20 terms of (**A**) BP, (**B**) MF, and (**C**) CC obtained by GO enrichment analysis. (**D**) KEGG pathway enrichment analysis results in the top 20 pathways. Abbreviations: BP, biological process; MF, molecular function; CC, cell composition.

We used GO and KEGG for a global evaluation of ketamine enantiomers, suicide, and depression overlapping genes. According to biological process (BP) analysis of GO, the top five terms were chemical synaptic transmission, anterograde trans-synaptic signaling, trans-synaptic signaling, synaptic signaling, and regulation of membrane potential (Figure 6A). Furthermore, based on fold enrichment, the one-carbon metabolic process, GABAergic synaptic transmission, and the GABA signaling pathway were also highlighted in BP (Figure 6A). The results of molecular function (MF) describing the top five terms were transmitter-gated ion channel activity, extracellular ligand-gated ion channel activity, carbonate dehydratase activity, gated channel activity, and ligand-gated ion channel activity (Figure 6B). Moreover, based on fold enrichment, NMDA glutamate receptor activity and GABA-gated chloride ion channel activity were also highlighted in MF (Figure 6B). The top five cellular components (CC) included synapse, neuron projection, somatodendritic compartment, synaptic membrane, and receptor complex (Figure 6C). According to CC, it was also possible to verify the importance of these genes as components of GABA-A receptor complex, GABA receptor complex, and NMDA selective glutamate receptor complex (Figure 6C). The top five signaling pathways according to the KEGG enrichment analysis were morphine addiction, nicotine addiction, neuroactive ligand-receptor interaction, Rap1 signaling pathway, and cAMP signaling pathway (Figure 6D). Furthermore, cocaine addiction, amphetamine addiction, GABAergic synapse, glutamatergic synapse, and dopaminergic synapse were also involved in the function of these genes (Figure 6D).

### 2.3. GO and KEGG Enrichment Analysis of Esketamine, Suicide, and Depression Overlapping Genes

**Figure 7:**
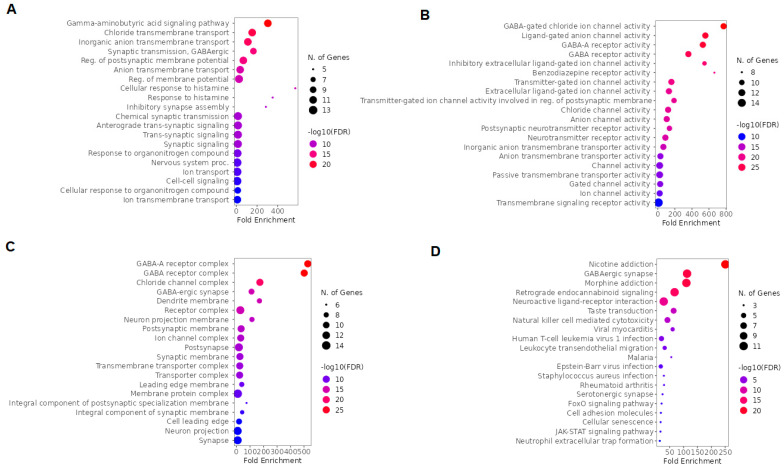
GO and KEGG enrichment analysis of esketamine, suicide, and depression-overlapping genes. The top 20 terms of (**A**) BP, (**B**) MF, and (**C**) CC obtained by GO enrichment analysis. (**D**) KEGG pathway enrichment analysis results in the top 20 pathways related. Abbreviations: BP, biological process; MF, molecular function; CC, cell composition.

We used GO and KEGG enrichment analysis to investigate the functions and biochemical pathways of esketamine, suicide, and depression overlapping genes. According to BP analysis of GO, the top five terms were GABA signaling pathway, chloride transmembrane transport, inorganic anion transmembrane transport, GABAergic synaptic transmission, and regulation of postsynaptic membrane potential (Figure 7A). Moreover, based on fold enrichment, cellular response to histamine, response to histamine, and inhibitory synapse assembly were highlighted in BP (Figure 7A). The top five MF terms were GABA-gated chloride ion channel activity, ligand-gated anion channel activity, GABA-A receptor activity, GABA receptor activity, and inhibitory extracellular ligand-gated ion channel activity (Figure 7B). Furthermore, benzodiazepine receptor activity demonstrated a high fold enrichment in MF (Figure 7B). The top five terms of CC were GABA-A receptor complex, GABA receptor complex, chloride channel complex, GABAergic synapse, and dendrite membrane (Figure 7C). According to KEGG enrichment analysis, the top 5 pathways were nicotine addiction, GABAergic synapse, morphine addiction, retrograde endocannabinoid signaling, and neuroactive ligand-receptor interaction (Figure 7D).

### 2.4. GO and KEGG Enrichment Analysis of Arketamine, Suicide, and Depression Overlapping Genes

**Figure 8:**
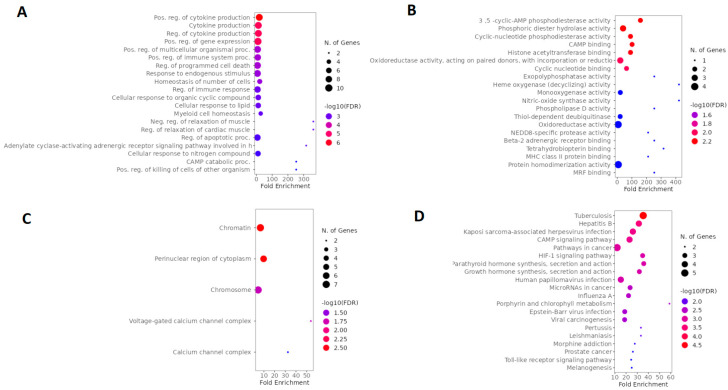
GO and KEGG enrichment analysis of arketamine, suicide, and depression overlapping genes. The top 20 terms of (**A**) BP, (**B**) MF, and (**C**) CC obtained by GO enrichment analysis. (**D**) KEGG pathway enrichment analysis results in the top 20 pathways. Abbreviations: BP, biological process; MF, molecular function; CC, cell composition.

To investigate the biological functions and biochemical pathways involved in the action of arketamine in depression and suicide, GO and KEGG analyses were performed. According to BP, the top five terms were positive regulation of cytokine production, cytokine production, regulation of cytokine production, positive regulation of gene expression, and positive regulation of multicellular organism processes (Figure 8A). Additionally, based on fold enrichment, negative regulation of relaxation of muscle, regulation of relaxation of cardiac muscle, adenylate cyclase-activating adrenergic receptor signaling pathway involved in heart process, cAMP catabolic process, and positive regulation of killing of cells of another organism were highlighted in BP (Figure 8A). The MF top five terms were 3′,5′-cyclic-AMP phosphodiesterase activity, phosphoric diester hydrolase activity, cyclic-nucleotide phosphodiesterase activity, 3′,5′-cyclic-nucleotide phosphodiesterase activity, and cAMP binding (Figure 8B). Furthermore, heme oxygenase activity, nitric-oxide synthase activity, and tetrahydrobiopterin binding demonstrated a high fold enrichment in MF (Figure 8B). The only significant cellular localizations in CC were chromatin, perinuclear region of cytoplasm, chromosome, voltage-gated calcium channel complex, and calcium channel complex (Figure 8C). The top 5 pathways indicated by KEGG were tuberculosis, hepatitis B, Kaposi sarcoma-associated herpesvirus infection, cAMP signaling pathway, and pathways in cancer (Figure 8D). These pathways are related to genes and proteins that are important in the immune response against infections and cancer.

### 2.5. GO and KEGG Enrichment Analysis of Overlapping Ketamine Enantiomers and Depression Genes

**Figure 9:**
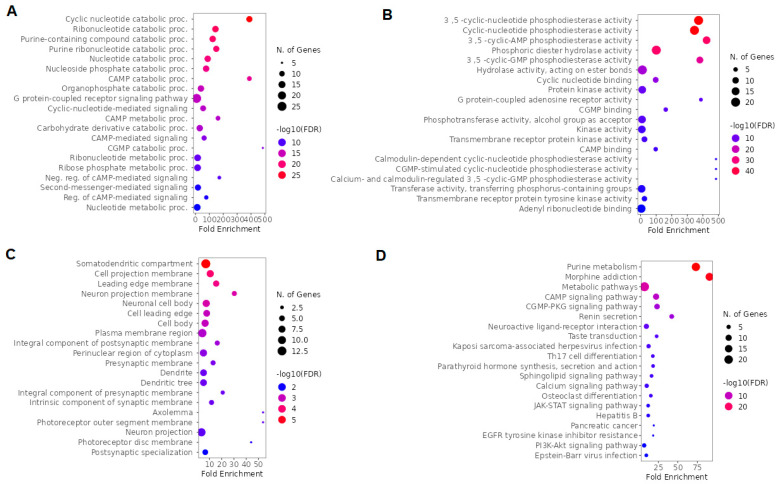
GO and KEGG enrichment analysis of overlapping ketamine enantiomers and depression overlapping genes. (**A**) BP, (**B**) MF, and (C) CC obtained by GO enrichment analysis (**D**) KEGG pathway enrichment analysis. Abbreviations: BP, biological process; MF, molecular function; CC, cell composition.

We used GO and KEGG for a global evaluation of overlapping ketamine enantiomers and depression genes. The top five BP terms were cyclic nucleotide catabolic process, ribonucleotide catabolic process, purine-containing compound catabolic process, purine ribonucleotide catabolic process, and cyclic nucleotide metabolic process (Figure 9A). Moreover, based on fold enrichment, the cAMP catabolic process and cGMP catabolic process were also highlighted in BP (Figure 9A). The MF top five terms were 3′,5′ -cyclic-nucleotide phosphodiesterase activity, cyclic-nucleotide phosphodiesterase activity, 3′,5′-cyclic-AMP phosphodiesterase activity, phosphoric diester hydrolase activity, and 3′,5′-cyclic-GMP phosphodiesterase activity (Figure 9B). The top five CC terms included somatodendritic compartment, cell projection membrane, leading edge membrane, neuron projection membrane, and neuronal cell body (Figure 9C). The top five pathways according to the KEGG enrichment analysis were purine metabolism, morphine addiction, metabolic pathways, cAMP signaling pathway, and cGMP-PKG signaling pathway (Figure 9D).

### 2.6. Molecular Docking

To perform docking, the targets of each filtered network were selected by the parameters DC and BC, plus the top five highest values of closeness centrality (CC). After selecting the top 5 biological targets from each network, molecular docking was performed according to the structures and parameters described in Table 1, which also shows the affinity values in kcal/mol. The results of the docking analyses revealed that, depending on the molecular target, there were differences in affinities between the ketamine enantiomers (Table 1). We selected those whose affinity values were within the range of −8.102 to −7.426 kcal/mol as priority interaction targets because they were values found for the GluN2B and GluN2A subunits of the NMDA receptor (encoded by *GRIN2B* and *GRIN2A* genes, respectively), which were consolidated pharmacological targets for both ketamine enantiomers, being considered our positive control in this docking study. Therefore, the main pharmacological targets of esketamine and the respective affinity values were GluN2B (−8.102 kcal/mol), PDE7A (−7.811 kcal/mol), PDE5A (−7.629 kcal/mol), JAK1 (−7.609 kcal/mol), PDE3A (−7.505 kcal/mol), GluN2A (−7.461 kcal/mol), and GABRG2 (−7.448 kcal/mol). The main pharmacological targets of arketamine and the respective affinity values were as follows: PDE3A (−8.120 kcal/mol), MIF (−8.008 kcal/mol), GluN2B (−7.974 kcal/mol), PDE7A (−7.826 kcal/mol), GABRB2 (−7.740 kcal/mol) PDE5A (−7.740 kcal/mol), JAK1 (−7.563 kcal/mol), and GluN2A (−7.426 kcal/mol). 

**Figure 10:**
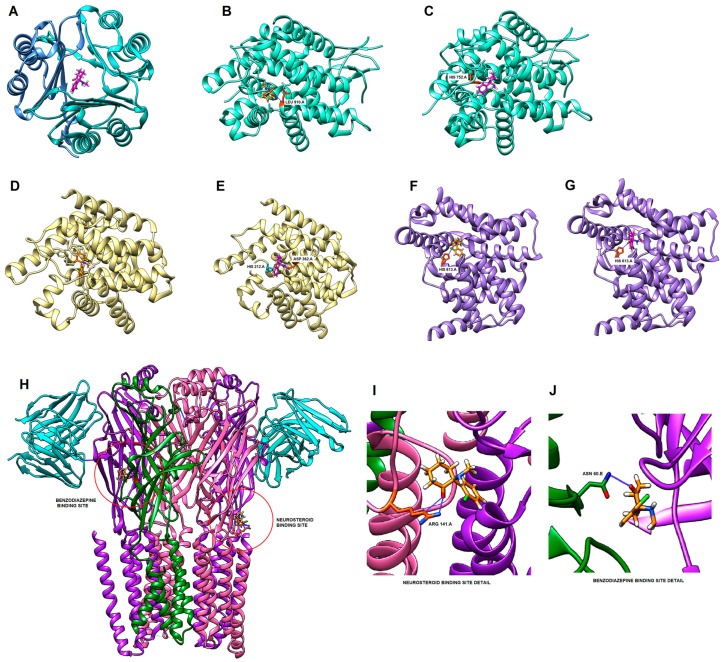
Molecular docking simulations (**A**) MIF and arketamine interacting in the center of protein. (**B**) PDE3A and esketamine in the X5M binding site. (**C**) PDE3A and arketamine in the X5M binding site. (**D**) PDE7A and esketamine in the IBM binding site. (**E**) PDE7A and arketamine in the IBM binding site. (**F**) PDE5A and esketamine in the 5GP binding site. (**G**) PDE5A and arketamine in the 5GP binding site. (**H**) Structure of 6D6T, which is a GABA_A_α1β2γ2 receptor, showing the neurosteroid (in left) and benzodiazepine (in right) binding sites. (**I**) GABA_A_α1β2γ2 receptor and esketamine in the neurosteroid binding site. (**J**) GABA_A_α1β2γ2 receptor and esketamine in the benzodiazepine binding site.

Figure 10 illustrates three-dimensional images from molecular docking simulations, indicating the interaction between the target proteins and the ketamine enantiomers at the respective binding sites and the presence or absence of hydrogen bonds. 

In the computational prediction, MIF (1GCZ structure) had one of the highest docking scores for arketamine (−8.008 kcal/mol) and interacted in the center of the protein, as illustrated in Figure 10A. 

According to docking scores, arketamine was predicted to have a higher affinity for PDE3A and PDE5A (−8.120 kcal/mol and −7.740 kcal/mol, respectively) than esketamine (−7.505 kcal/mol and −7.629 kcal/mol, respectively), while the affinity of esketamine and arketamine was similar for PDE7A (−7.811 kcal/mol and −7.826 kcal/mol, respectively). In molecular docking simulations, we found hydrogen bond interactions between PDE3A Leu910 and esketamine (Figure 10B), PDE7A Asp362 and arketamine (Figure 10D), PDE7A His212 and arketamine (Figure 10E), PDE5A His613 and esketamine (Figure 10F), and PDE5A His613 and arketamine (Figure 10G). No hydrogen bridges were found between PDE3A and arketamine (Figure 10C).

We also found that the *GABRB2*, *GABRB3*, and *GABRA3* genes were potential targets shared by both enantiomers of ketamine. In the molecular docking analyses, the proteins obtained from PDB were 6D6T (GABA_A_α1β2γ2 receptor), 6DW1 (GABA_A_α1β1γ2 receptor), and 7QNA (GABA_A_α4β3γ2 receptor). 

According to computational predictions, ketamine enantiomers had a higher affinity for the 6D6T structure than 6DW1 and 7QNA structures. In addition, the chloride channel (−7.747 kcal/mol for arketamine), the benzodiazepine site (−7.448 kcal /mol for esketamine), and the neurosteroid site (−7.371 kcal/mol for esketamine and −7.387 kcal/mol for arketamine) were potential targets for interaction with ketamine. Figure 10H illustrates the 6D6T structure, which is a GABA_A_α1β2γ2 receptor, showing the neurosteroid (left) and benzodiazepine (right) binding sites. In molecular docking simulations, we found two hydrogen bond interactions between GABA_A_ receptor Arg141 and esketamine in the neurosteroid binding site (Figure 10I), and a hydrogen bond between GABA_A_ receptor Asn60 and esketamine in the benzodiazepine binding site (Figure 10J).

## 3. Discussion

The main purpose of this study was to explore the potential molecular targets of ketamine enantiomers in suicide and MDD by employing network pharmacology and molecular docking simulations. Our workflow involved a sequential analysis composed of the following steps: (a) identification of molecular targets of ketamine enantiomers in SwissTargetPrediction; (b) collection of genes targets of MDD and suicide in GeneCards; (c) creation of a Venn diagram to obtain the overlapping genes; (d) construction of the network of overlapped genes; (e) implementation of network filter parameters to obtain the highest closeness, betweenness, and degree centrality; and (f) running molecular docking simulations. Our bioinformatics analysis uncovered that both enantiomers of ketamine, esketamine, and arketamine share biological targets that may be implicated in their antidepressant and antisuicide properties. However, due to the large number of pharmacological targets found in the network analyses, we limited the molecular docking analyses to the nodes that had the best topological parameters. The main targets obtained are NMDA receptors (GluN2 subunits), GABA receptors (GABRA3, GABRB2, GABRB3 subunits), macrophage migration inhibitory factor (MIF), and various subtypes of phosphodiesterases (PDE3A, PDE7A, PDE5A). However, it is important to consider that other targets not analyzed may be relevant to the antidepressant and antisuicidal effects of ketamine enantiomers.

GO and KEGG functional analyses pointed out relevant targets for both ketamine enantiomers and were useful in directing subsequent studies. In GO enrichment analysis related to molecular function, NMDA receptor activity was pointed out as one of the main targets. In agreement, ketamine is a well-known, noncompetitive open-channel NMDAr antagonist [8]. The GRIN gene family encodes three classes of NMDA receptor (NMDAR) subunits: glycine-binding GluN1 (product of *GRIN1*), glutamate-binding GluN2 (*GRIN2A, GRIN2B*, *GRIN2C*, and *GRIN2D*), and glycine-binding GluN3 subunit (*GRIN3A* and *GRIN3B*). Regarding GluN2, four different subunits (GluN2A-D) are present throughout the central nervous system [22]. Of note, NMDA receptors are composed of two obligatory GluN1 subunits, and a combination of two additional GluN subunits (GluN2A-D, GluN3A, B) [23].

We found that GluN2 subunits of NMDAr are potential targets for the action of both ketamine enantiomers, particularly GluN2B, using docking analyses. Interestingly, a study by Zhang and co-workers [9] reported that both enantiomers of ketamine interact at NMDAr in the transmembrane portion of the channel at key amino acid residues, such as Leu642 in GLuN2A, Leu643 in GluN2B, and Asn616 in GluN1. These amino acid residues form hydrophobic interactions and hydrogen bonds with ketamine, and mutations in these residues can decrease the potency of ketamine in blocking the NMDAr. Interestingly, several studies have indicated that the antagonism of ketamine at NMDAr is implicated in its rapid antidepressant response, particularly at the NR2B subunit [24,25]. The genetic deletion of GluN2B from cortical neurons caused a marked antidepressant-like effect in mice. In addition, ketamine was devoid of an antidepressant-like effect in the tail suspension test in the animals that had such GluN2B deletion [24]. Further reinforcing the role of GluN2B in the antidepressant effect of ketamine, a study by Tang et al. (2020) [26] showed that CaMKIIα affects phosphorylation and extrasynaptic localization of GluN2B. Indeed, ketamine was proposed to selectively block extra-synaptic GluN2B-containing NMDARs, causing a subsequent activation of mTORC1, which in turn induces protein synthesis [27]. The docking analyses indicated a slightly higher affinity of the esketamine enantiomer at GluN2B than arketamine. 

Although NMDAr is the key target of esketamine and arketamine, other targets may be implicated in their antidepressant properties. Indeed, it has been discussed whether the inhibition of NMDAR is critical for the mood-enhancing effects of ketamine enantiomers, and a hypothesis that has been raised is that their antidepressant and antisuicidal effects may be dependent on their interaction with multiple molecular targets [28]. Regarding this issue, our study may contribute to identifying potential protein targets.

Network construction of overlapping arketamine, suicide, and depression genes identified macrophage migration inhibitory factor (*MIF*) as a central node. Subsequently, our study identified MIF, to our knowledge, for the first time, as a potential molecular target of arketamine by molecular docking. MIF is a cytokine originally characterized by its ability to prevent migration of macrophages, which is now recognized as a key mediator of the innate immune system and adaptive immunity [29]. It is a glycoprotein expressed in many different types of cells and tissues, including brain regions, such as the hippocampus and cerebral cortex (regions implicated in mood regulation) [30,31]. The release of MIF by either anterior hypophysis or monocytes/macrophages, is stimulated by inflammatory stimuli and/or stress [29]. More recently, evidence has pointed out that MIF is involved in the assembly and activation of the NLRP3 inflammasome [32]. The role of MIF in MDD has been increasingly investigated in recent years. The genetic deletion of MIF caused anxiety- and depression-like behavior and impairments in hippocampal-dependent memory in mice [30]. Interestingly, the blood levels of MIF have been shown to be increased in patients with MDD [33]. In a study carried out with healthy university undergraduates, MIF levels were 40% higher in individuals who presented high depressive symptoms as compared to those with low depressive symptoms [34]. In addition, MIF levels were higher in depressive patients who later failed to respond to antidepressants compared with those responsive to treatments [35]. Despite these data implicating MIF in the pathophysiology of MDD, the effects of ketamine on MIF levels, both in clinical studies and in animal models of depression, have not been explored, deserving future studies. The role of MIF in the neurobiology of suicide also needs to be further explored, since it has not been well established. While the study by Aytak et al. in 2020 [36] found that *MIF* gene polymorphism may be related to attempted suicide in patients with bipolar disorder, other studies failed to indicate a relationship between MIF levels or *MIF* gene polymorphism and suicide [37,38]. The study by Shimmyo et al. [37] did not find a higher risk of suicide in a Japanese population with the *MIF*-794CATT5–8 microsatellite or *MIF*-173G/C single-nucleotide polymorphism (SNP) on the *MIF* gene promoter. In addition, no significant alterations in MIF levels in the dorsolateral prefrontal cortex were observed in suicide completers as compared to control individuals [38]. 

Another interesting finding of the bioinformatic analysis is that 3′,5′-cyclic nucleotide phosphodiesterases (PDE) may be molecular targets of ketamine enantiomers. PDEs are classified into 11 subtypes based on sequence homology and structural and enzymatic features mainly related to sensitivity to modulators [39]. These enzymes are responsible for the hydrolysis of the intracellular second messengers cyclic adenosine monophosphate (cAMP) and/or cyclic guanosine monophosphate (cGMP), which are essential for synaptic plasticity, learning, and memory [40]. In addition, they play an important role in mood regulation [41]. Network pharmacology indicated the possible implication of several *PDE* genes as targets for ketamine enantiomers in MDD, as indicated by the high degree of clustering. Although other *PDEs* have been indicated by the network pharmacology approach, we selected four isoforms considering the highest centrality parameters obtained in the network analysis. Subsequently, based on the binding energy of the protein-ligand complex, PDE3A, PDE7A, and PDE5A were chosen for the tridimensional visualization and analysis of hydrogen bonds. In particular, both esketamine and arketamine have high affinity for these PDE isoforms. 

PDE3 occurs as two groups of isoenzymes (PDE3A and 3B) that are involved in the hydrolysis of cGMP, mainly cAMP [42,43]. Although the role of PDE3A in the pathophysiology of MDD is not well established, this enzyme may be implicated in depressive symptoms, since cilostazol, a PDE 3A inhibitor, has been reported to decrease the severity of depressive symptoms assessed by Hamilton depression rating scale scores in poststroke depression patients [44].

PDE7 comprises 2 subclass members: PDE7A and B. This enzyme, abundant in the brain, is responsible for the hydrolysis of cAMP. The highest levels of PDE7A have been reported in the olfactory bulb, olfactory tubercle, hippocampus, cerebellum, medial habenula nucleus, pineal gland, area postrema, and choroid plexus [45]. Notably, PDE7A inhibitors have been shown to exhibit procognitive and antidepressant properties [46]. 

PDE5 hydrolyzes cGMP, which is a critical component of the cGMP-protein kinase G (PKG) intracellular signaling in neurons. There are three isoforms of PDE5A, encoded by a unique gene (*PDE5A* gene), namely PDE5A1, PDE5A2, and PDE5A3 [47]. It has been reported that the *PDE5A* single nucleotide polymorphism (SNP) may be associated with MDD [48]. The involvement of PDE5A in MDD is reinforced by the antidepressant-like effect elicited by the PDE5A inhibitor sildenafil in chronic unpredictable mild stress [49].

Considering the involvement of the above-mentioned PDE in the pathophysiology of MDD, the possible role of ketamine in the modulation of the activity of these enzymes is poorly explored and needs to be further investigated.

Network pharmacology, GO, KEGG analysis, and molecular docking pointed out that GABA genes are also potential targets for both enantiomers but mainly for esketamine. The GABA receptor (GABAr) family is divided into metabotropic and ionotropic receptors. GABA_B_r are heterodimeric metabotropic receptors and are associated with potassium and calcium channels via G protein. GABA_B_ receptors are members of the class C G protein-coupled receptor (GPCR) family. These receptors have R1 subunits where orthostatic ligands bind (encoded by the *GABBR1* gene) and R2 coupled to G protein (*GABBR2*) [50]. GABA_A_r are pentamer chloride channels and have 19 genes that encode for subunits of the GABA_A_ receptor, which is organized into classes (α, β, γ, ρ, θ, ε, π and δ) and isoforms (α1–6, β1–3, γ1–3 and ρ1–3) [51]. Thus, the *GABRA1* gene encodes the GABA Type A Receptor Subunit Alpha1(GABA_A_α1) protein, the *GABRB3* gene has GABA Type A Receptor Subunit Beta3 (GABA_A_β3) as its product, and the *GABRG2* gene encodes the GABA Type A Receptor Subunit Gamma2 (GABA_A_G2) protein. The assembly of GABA_A_r as heteropentamers produces complex heterogeneity in their structure, and different subunit compositions are possible. A typical GABA_A_ receptors are made up of two alpha subunits, two beta subunits, and one gamma subunit, and the interaction sites lie at the connections between these subunits. The GABA interaction site is located between the interfaces of the alpha and beta subunits, the neurosteroid site is located in the transmembrane domain between the alpha and beta subunits, and the benzodiazepine site is located at the interface between the alpha and gamma subunits [52]. In our study, we found that the *GABRB2*, *GABRB3*, and *GABRA3* genes are potential targets shared by both enantiomers of ketamine, while GABA_A_G2 coded by *GABRG2* is predicted to mainly interact with esketamine. 

The benzodiazepine site of GABAr is closely implicated in anhedonic behavior. Anhedonia is one of the symptoms of major depressive disorders that is closely related to suicide risk [53]. Of note, ketamine has been reported to attenuate symptoms of anhedonia, and this property might be implicated in its ability to prevent suicidal ideation [54]. A study investigating MRK-016 (a negative allosteric modulator of GABA_A_r) reported that this compound recovered hedonic behavior in mice and that this behavior was prevented using the benzodiazepine antagonist flumazenil, suggesting the involvement of the benzodiazepine site of GABA_A_r in anhedonia symptoms [55]. A clinical study showed that esketamine improved anhedonic symptoms in patients with unipolar and bipolar depression [56]. These data support the computational prediction that the benzodiazepine site of GABA_A_r may be involved in the mechanism of action of ketamine in improving anhedonic symptoms in MDD.

Neurosteroid analogs, molecules that bind to the allosteric site of neurosteroids on GABA_A_r, have potential use as antidepressants and anesthetics [57]. Reinforcing the role of GABA_A_ receptor to rapid antidepressant responses, brexanolone and zuranolone, positive allosteric modulators of the neurosteroid site of this receptor elicit rapid and sustained antidepressant effects in postpartum depression and MDD [58,59,60]. 

Ketamine is known for its dissociative properties and abuse potential [61], used even as an animal model of schizophrenia at doses higher than the ones that elicit antidepressant effects [62]. In agreement, the KEGG and GO analyses indicated that both enantiomers have the potential to activate addiction-related pathways, such as nicotine, morphine, amphetamine, and cocaine addiction, due to the involvement of GABAergic and glutamatergic receptors. On the other hand, ketamine has the potential to treat many addictions [63], requiring further research in this field. 

One of the limitations of our study is that we cannot predict the effect of gender on the findings for each ketamine enantiomer. The availability of software able to predict sex differences between esketamine and arketamine enantiomers in depression and suicide would be welcome to provide information on this issue. However, it has been reported in preclinical studies that females are more sensitive to the antidepressant effect of ketamine [64,65,66], but males have a prolonged response [67]. Regarding S-ketamine, a recent study did not find any sex-specific or estrous cycle-specific differences in antidepressant-like responses to esketamine in a genetic animal model of depression (Flinders Sensitive Line rats) [68]. In a similar manner, no sex differences were observed in a mouse model of inflammation (LPS) in animals treated with arketamine [69]. In clinical studies, no significant differences in ketamine treatment response have been reported between men and women, but gender differences in side effects, metabolization, and time-related effective doses of ketamine seem to exist [70]. Therefore, further in silico, preclinical, and clinical studies are needed to investigate the antidepressant and antisuicidal effects of ketamine enantiomers in males and females.

Although our study suggests novel and poorly explored proteins as possible targets for the antidepressant and antisuicidal effects of ketamine enantiomers, future experimental studies are needed to confirm the role of these targets in the effects of ketamine enantiomers. 

## 4. Materials and Methods

### 4.1. Identification of the Potential Target Genes of Ketamine Enantiomers in Suicide and Depression

The list of biological targets of arketamine and esketamine was obtained from SwissTargetPrediction (Swiss Institute of Bioinformatics, Lausanne, Switzerland) [71] by searching the structure of ketamine (Pubchem CID 3821) [72], with the selection of the enantiomers using the “calculate stereo” tool by Marvin JS. The lists of genes related to depression and suicide were obtained from the GeneCards database (Weizmann Institute of Science, Rehovot, Israel) [73] by searching for the words “depression” and “suicide”, respectively. The searches were carried out on 8 December 2022. Using Venny v. 2.1.0 (Centro Nacional de Biotecnología, Madrid, Spain), we obtained the overlapping target genes between the groups.

### 4.2. Gene–Gene Interaction Network Construction and Analysis

The list of genes obtained from the crossover was searched in GeneMANIA (University of Toronto, Toronto, Canada) using default settings [74], and a network was obtained. The search was performed only in *Homo sapiens*. The network was imported and analyzed in Cytoscape V. 3.9.1 [75]. Topological network analysis was performed to identify the most important nodes (genes) using degree centrality (DC), betweenness centrality (BC), and closeness centrality (CC) criteria, whose cut-off value was defined according to each network’s characteristics. CC, DC, and BC are variables based on independence, connectivity, and control in the network, respectively, and describe distinct topological properties in the network, which, in combination, may be relevant for selecting more important biological targets [76]. It is common practice to select/filter targets from network pharmacology for molecular docking that obtain good scores of at least these three characteristics [14,16,77]. The filters were applied to the already computed model.

### 4.3. GO and KEGG Enrichment Analysis

GO and KEGG enrichment analyses were performed in ShinyGO v.0.76.3 (South Dakota State University, Brookings, USA) [78] for each network built in GeneMania and Cytoscape. In this analysis, the terms biological process (BP), molecular function (MF), and cellular composition (CC) were used for GO and KEGG, and using the organism Homo sapiens, false discovery rate (FDR) <0.05 and 20 most important pathways as filters. The lowest FDR values were used to select the most important KEGG biochemical pathways or GO analyses.

### 4.4. Molecular Docking

The molecular docking analysis was carried out to predict the binding affinity between arketamine or esketamine and the potential biological targets using the DockThor 2.0 software (LNCC, Petrópolis, Brazil) [79] in a similar way as described previously [80]. The protein database (RCSB—Protein Data Bank) [81] was used to obtain the structures of biological targets and the ligand structures of esketamine (JC9) and arketamine (RKE). All binders underwent geometric optimization using Chimera 1.14 software 0 (UCSF, San Francisco, CA, USA) [82]. The preparation of target proteins included the removal of water molecules, the addition of hydrogens, and the assignment of Gasteiger charges. In some structures, it was necessary to delete the ligands/antagonists/agonists present at the binding site, as well as chains or parts of chains that were not part of the grid box, to optimize the docking analysis. The PDB structures used and the binding sites are described in Table 1. Additionally, the poses of ligands were also observed using Chimera 1.14.

## 5. Conclusions

In summary, our study uncovered that ketamine had a polypharmacological profile, with many biological targets involved in suicide and MDD. The findings indicated GRIN2A, GRIN2B, PDE7A, PDE5A, and PDE3A as the most likely targets predicted for both ketamine enantiomers. Regarding S-ketamine, it was predicted that the main targets were GABRB2 and GABRG2. In the case of arketamine, it was predicted that MIF was the main target. These new targets pointed out in our study have not yet been explored with regard to their involvement in the antidepressant and antisuicidal effects of ketamine and therefore deserve attention in future experimental studies.

## Figures and Tables

**Table 1:** Ketamine’s Top 20 Targets Based on Docking Scores.

Gene Symbol	PDB ID	Ligand ID (Binding Site)	Esketamine Docking Score (kcal/mol)	Arketamine Docking Score (kcal/mol)
*GRIN2B*	7EU8	JC9 (ketamine)	−8.102	−7.974
*PDE3A*	7KWE	X5M	−7.505	−8.120
*MIF*	1GCZ	center of protein	N.A.	−8.008
*PDE7A*	1ZKL	IBM	−7.811	−7.826
*PDE5A*	1T9S	5GP	−7.629	−7.740
*GABRB2*	6D6T	Cl channel	N.A.	−7.747
*JAK1*	4E4N	0NL	−7.609	−7.563
*GABRG2*	6D6T	FYP (benzodiazepines)	−7.448	N.A.
*GRIN2A*	7EU7	JC9 (ketamine)	−7.461	−7.426
*GABRB2*	6D6T	Y01 (neurosteroids)	−7.371	−7.387
*PDE4D*	1OYN	ROL	N.A.	−7.347
*CREB1*	5ZKO	center of protein	N.A.	−7.339
*EGFR*	7OXB	35Z	−7.117	−6.986
*CD74*	5KSV	Between alpha helices and beta sheets	N.A.	−7.038
*F13A1*	5MHL	MI0621	−7.032	−6.931
*GABRA4*	7QNA	ABU (GABA)	−7.027	N.A.
*ACTN4*	6OA6	center of protein	N.A.	−7.004
*GABRB1*	6DW1	ABU (GABA)	−6.720	N.A.
*GABRA1*	6D6T	ABU (GABA)	−6.608	N.A.
*GABRB2*	6D6T	ABU (GABA)	−6.608	−6.465
*GRM4*	7E9H	SEP (phosphoserine)	−6.322	−6.308

N.A. not analyzed.

## Data Availability

Data is contained within the article.
